# CD73/adenosine axis exerts cardioprotection against hypobaric hypoxia-induced metabolic shift and myocarditis in a sex-dependent manner

**DOI:** 10.1186/s12964-024-01535-8

**Published:** 2024-03-07

**Authors:** Marie Louise Ndzie Noah, Richard Mprah, Prosperl Ivette Wowui, Adebayo Oluwafemi Adekunle, Joseph Adu-Amankwaah, Rubin Tan, Zheng Gong, Tao Li, Lu Fu, Jeremiah Ong’achwa Machuki, Shijie Zhang, Hong Sun

**Affiliations:** Department of Physiology, Xuzhou Medical University, 209 Tongshan Road, Xuzhou, Jiangsu 221004 China

**Keywords:** Sex differences, Metabolic shift, Inflammation, CD73/adenosine axis, Hypobaric hypoxia, Cardiac dysfunction

## Abstract

**Background:**

Clinical and experimental studies have shown that the myocardial inflammatory response during pathological events varies between males and females. However, the cellular and molecular mechanisms of these sex differences remain elusive. CD73/adenosine axis has been linked to anti-inflammatory responses, but its sex-specific cardioprotective role is unclear. The present study aimed to investigate whether the CD73/adenosine axis elicits sex-dependent cardioprotection during metabolic changes and myocarditis induced by hypobaric hypoxia.

**Methods:**

For 7 days, male and female mice received daily injections of the CD73 inhibitor adenosine 5′- (α, β-methylene) diphosphate (APCP) 10 mg/kg/day while they were kept under normobaric normoxic and hypobaric hypoxic conditions. We evaluated the effects of hypobaric hypoxia on the CD73/adenosine axis, myocardial hypertrophy, and cardiac electrical activity and function. In addition, metabolic homeostasis and immunoregulation were investigated to clarify the sex-dependent cardioprotection of the CD73/adenosine axis.

**Results:**

Hypobaric hypoxia-induced cardiac dysfunction and adverse remodeling were more pronounced in male mice. Also, male mice had hyperactivity of the CD73/adenosine axis, which aggravated myocarditis and metabolic shift compared to female mice. In addition, CD73 inhibition triggered prostatic acid phosphatase ectonucleotidase enzymatic activity to sustain adenosine overproduction in male mice but not in female mice. Moreover, dual inhibition prostatic acid phosphatase and CD73 enzymatic activities in male mice moderated adenosine content, alleviating glycolytic shift and proinflammatory response.

**Conclusion:**

The CD73/adenosine axis confers a sex-dependent cardioprotection. In addition, extracellular adenosine production in the hearts of male mice is influenced by prostatic acid phosphatase and tissue nonspecific alkaline phosphatase.

**Supplementary Information:**

The online version contains supplementary material available at 10.1186/s12964-024-01535-8.

## Introduction

Providing oxygen to organs, tissues, and cells in high-altitude environments presents a significant challenge. It has been reported that individuals residing or visiting regions above 2500 m frequently experience altitude sickness, characterized by shortness of breath, headache, dizziness, fatigue, confusion, and loss of appetite [1, 2]. Also, the role of hypoxia in the pathophysiology of various diseases, notably cardiovascular diseases (CVDs), has been demonstrated [3]. During most CVDs, the supply of oxygen and nutrients decreases or stops entirely, triggering several preservative mechanisms such as increased oxygen transport and delivery and increased blood flow to restore the cells to a normoxic state [4]. The failure to restore normoxia may drive increased proinflammatory cytokines (IL-1β and IL-6, IL-18) production and metabolic switch from fatty acid oxidation to glycolysis, resulting in cardiac hypertrophy, myocarditis and ultimately heart failure [4–6].

It is widely known that men are more likely than women of the same age to develop CVDs [7]. However, the incidence of CVD increases in women after menopause [8]. Males are known to exhibit a higher susceptibility to developing dilated cardiomyopathy or heart failure due to impaired cardiac remodeling and stress-induced cardiac responses [9, 10]. Barcena et al. highlighted a sex-specific variation in inflammatory response, noting a proinflammatory phenotype in males and an anti-inflammatory phenotype in females [11]. They reported that both phenotypes significantly impact heart function in autoimmune myocarditis [11].

CD73, a glycosylphosphatidylinositol (GPI)-linked membrane-bound glycoprotein, has been reported to be a cardioprotective protein. CD73 and its upstream CD39 enzymatic activity are critical in regulating the duration, amplitude, and chemical nature of purinergic signals via the conversion of ADP/ATP to AMP and AMP to adenosine [12]. CD73 plays a significant role in CVDs as an essential element of the extracellular adenosinergic axis [13, 14]. Adenosine, formed by CD73, interacts with purinergic receptors (A_1_AR, A_2A_AR and A_2B_AR, and A_3_AR) coupled to G proteins and acts via specific transporters to induce various responses (contraction, cell growth control, tissue remodeling, and immune-metabolic regulation) in the heart [7, 12, 15, 16]. It has also been reported that CD73-derived adenosine may promote tissue adaptability or reduce hypoxia-induced inflammation [17, 18]. Also, Quast et al. demonstrated that adenosine formed by CD73 inhibits cardiac inflammation and fibrosis in transverse aortic constriction (TAC)-induced heart failure [14]. Recent studies have shown a sex-specific immuno-metabolic and neuromodulatory response of the CD73/ adenosine axis in the brain and liver [19, 20]. However, the sex difference on the CD73/ adenosine axis during cardiac dysfunction is poorly elucidated. Hence, we hypothesize that the CD73/adenosine axis may play a sex-dependent cardioprotective function during hypobaric hypoxia (HH).

To check our hypothesis, the effects of HH on the CD73/adenosine axis, myocardial hypertrophy, and cardiac electrical activity and function were evaluated in male and female mice. In addition, metabolic homeostasis and immunoregulation were assessed to clarify the sex-dependent cardio-protection of the CD73/adenosine axis.

## Materials and methods

### Pharmacological agents

CD73 inhibitor Adenosine 5′-(α, β-methylene) diphosphate sodium salt (APCP) was purchased from Tocris Bio-Techne, and the prostatic acid phosphatase (PAP) inhibitor – Benzylphosphonic acid (GB57232) (BPA) was purchased from GlpBio Technology Inc. All the drugs were prepared according to the manufacturer's instructions.

### Experimental animals and models

Male (M) and female (F) wild-type FVB (8—12-week-old) mice received a daily intraperitoneal (i.p) injection of 10 mg/kg/day of adenosine 5′- (α, β-methylene) diphosphate (APCP) for 7 consecutive days as previously reported [21]. The mice were divided into normobaric normoxic (NN) and hypobaric hypoxia (HH) groups. HH mice were kept and fed in a hypobaric hypoxia chamber (Guizhou Fenglei Aviation Machinery Co., Ltd., Guizhou, China: FLYDWC50-IIA), where the HH environment was generated by increasing the altitude to 3000 m (14.8 kPa and 13.36% O_2_) for 10 min, then 4500 m (12.1 kPa and 11.8%O_2_) for 10 min, 5500 m (10.5 kPa and 10.5%O_2_) for 20 min, and finally increasing to 6000 m (10.0 kPa and 9.92%O_2_) altitude for 7 consecutive days. In contrast, mice in the NN group were kept and fed in an NN environment at sea level (ambient oxygen levels) for 7 consecutive days.

To explore the therapeutic potential of double inhibition of CD73 and PAP, only male mice received a single i.p injection of 5 mg/kg/day of BPA, APCP and a double injection of APCP and BPA consecutively for 7 days (the dosage of BPA was determined according to previous reports [22, 23]). The mice were divided into groups of NN (BPA and APCP + BPA) and HH (BPA and APCP + BPA). The HH (BPA and APCP + BPA) were, in a stepwise manner, exposed to HH while receiving APCP and BPA treatment. At the end of the animal model, mice were removed from the HH chamber and were immediately anesthetized with esketamine hydrochloride for electrocardiography assessment. Afterward, the hearts were harvested via thoracotomy, wet-weighed immediately for morphometric assessment, and processed for further analysis. In addition, another batch of mice was used for echocardiography assessment.

### Electrocardiography and Echocardiography

Electrocardiography (EKG) was performed on mice at the end of the model using PowerLab systems' 3-lead monopolar needle electrode (ADInstruments), and echocardiography was performed as previously described [24, 25] using Silicon wave60 (KOLO Medical). M-mode images were taken, and the functional parameters were calculated using the left ventricular systolic and diastolic diameters.

### Enzyme-linked immunosorbent assay (ELISA)

Myocardia lysates were used to examine the concentration of proinflammatory IL-1β (JL18442; Jianglai Bio. Tech), TNF-α (JL10484; Jianglai Bio. Tech.), anti-inflammatory biomarkers (IL-10 (JL20242; Jianglai Bio. Tech.), TGF-β (JL13959; Jianglai Bio. Tech.), CD73 (JL13102; Jianglai Bio. Tech.) and cardiac hypertrophy/injury markers ANP (EK12636; Sabbiotech.), and BNP (EK12906; Sabbiotech.). Sera were used to assess cardiac troponin I (cTnI) (EK1821; Sabbiotech.). ELISAs were done in triplicates according to the manufacturer's instructions.

### Adenosine assay

Following the manufacturer's instructions, fluorometric assay (MET-5090, Cell BioLabs) was used to measure the myocardial lysate's adenosine concentration (*n* = 4/group). A microplate reader was used to measure the amount of adenosine content at 570 nm**.**

### Histology, Immunohistochemistry (IHC), Immunofluorescence (IF) and Biochemical staining

#### Wheat germ agglutinin (WGA) staining

Cryopreserved heart slices were fixed in 4% formaldehyde for 30 min at room temperature (RT), followed by three PBS washes and a 15-min HBSS priming period. Afterward, the cardiac slices were stained with WGA (Thermo Fisher Scientific; W11261) and left in the dark for 10 min at room temperature, followed by PBS washes and DAPI counterstaining. The cardiomyocyte surface area was measured using a fluorescence microscope at X60 magnification and computed using ImageJ (1.52a version; National Institute of Health USA).

#### IHC and IF staining

IHC staining for CD86 (Abcam; ab53004; 1:1000); CD206 (Abcam; ab8918; 1:1000) and IF staining for Glut1 (E4S6I) (Rabbit mAb #73,015) and F4/80(C-7): sc-377009) was performed as previously described [26], with some modifications. Frozen sections were used; therefore, the original experiment's antigen retrieval phase was skipped, and myocardial slices were fixed in 4% formaldehyde for 15 min before staining. CD86 + and CD206 + were detected using a light microscope (Olumpus CX31) at X40 magnification. Also, Glut1 and F4/80 were detected using a fluorescence microscope at X60 magnification. They were then quantified using ImageJ (1.52a version; National Institute of Health USA).

#### Oil Red O (ORO) staining

Myocardial lipid depositions were assessed to investigate the metabolic condition of the hearts using the ORO staining kit (Solarbio; G1261) according to the manufacturer's instructions. ImageJ was used to quantify lipid depositions detected at X40 magnification.

#### Periodic Acid Schiff (PAS) staining

The myocardia's glycogen and other polysaccharide contents were evaluated using the PAS staining kit (Solarbio; G1281) according to the manufacturer's instructions. ImageJ was used to identify and quantify PAS-positive areas detected at X40 magnification.

### Western blot

To extract proteins, hearts were washed with cold PBS homogenized, and then cocktails of RIPA, protease, and phosphatase inhibitors (ratio 100:1:1) were added. Protein concentrations were standardized before being electrophoresed on 10–12% gels and transferred to a 0.45 m PVDF membrane (Millipore Immobilon®-P; IPVH08100). Membranes were blocked in TBST with 2% BSA, and proteins of interest were blotted with the antibodies listed here: Glut1 (E4S6I) (Rabbit mAb #73015; CD36 (NB400-144) Novus Biologicals; 1:2000); Cleavage Caspase 3 (Asp175) (D3E9); Cell Signaling Technology; #9579 Rabit mAb;1:1000), CD73 (Abcam; Ab175396; 1:1000), anti-GATA4 (Abcam; ab84593; 1:1000), TNAP (ALPL) Rabbit mAb (A0514); PAP (PSAP) Rabbit mAb (A21195); GAPDH (Proteintech; 10494–1-AP; 1:1000) and HRP-conjugated Goat Anti-Rabbit IgG(H + L) (Proteintech; SA00001-2; 1:1000). Enhanced chemiluminescence (Tanon, China) was used to visualize the membranes.

### Quantitative RT-PCR

mRNAs were extracted from cardiac tissue using TRIzol™ Reagent (Invitrogen™; 15596026), cDNAs were generated using a reverse transcription kit (FSQ107; Toyobo), and qPCR analysis was performed using SYBR Green Master Mix (Q111-02; Vazyme) according to manufacturer's instructions. The adenosine receptors (A1, A2b, A2a, and A3) and IL-18 genes were measured using the primer sequences in Table 1. The housekeeping gene GAPDH was used, and mRNA fold changes were calculated using the 2Ct technique.

**Table 1:** List of primer sequences used for RT-qPCR

No	Gene	Antisense	Sense
1	Adenosine A1	TGTGCCCGGAAATGTACTGG	TCTGTGGCCCAATGTTGATAAG
2	AdenosineA2b	ATGGCAGCTAGAGACGCAAGAC	GGGATACCAGAAAGTAGTTGGTG
3	AdenosineA2a	GCAATAGCCAAGAGGCTGAAGA	GCCATCCCATTCGCCATCA
4	Adenosine A3	AAGGTGAAATCAGGTGTTGAGC	AGGCAATAATGTTGCACGAGT
5	IL-18	CCTACAATGTTACAACACTTAAAAT	ATAAGCCCTAAATATATGTATCCTTA
6	GAPDH	TGTGATGGGTGTGAACCACGAGAA	AGTGATGGCATGGACTGTGGTCAT

### Statistics

GraphPad Prism 5.0 (San Diego, CA, USA) was used for statistical analysis. All data are presented as Mean ± SEM, and grouped data were analyzed using two-way ANOVA. Tukey's multiple comparisons test was used to determine whether post-hoc analyses were statistically significant (*p* < 0.05).

## Results

### HH induced alteration of cardiac function with a male predominance in severity

**Fig. 1:**
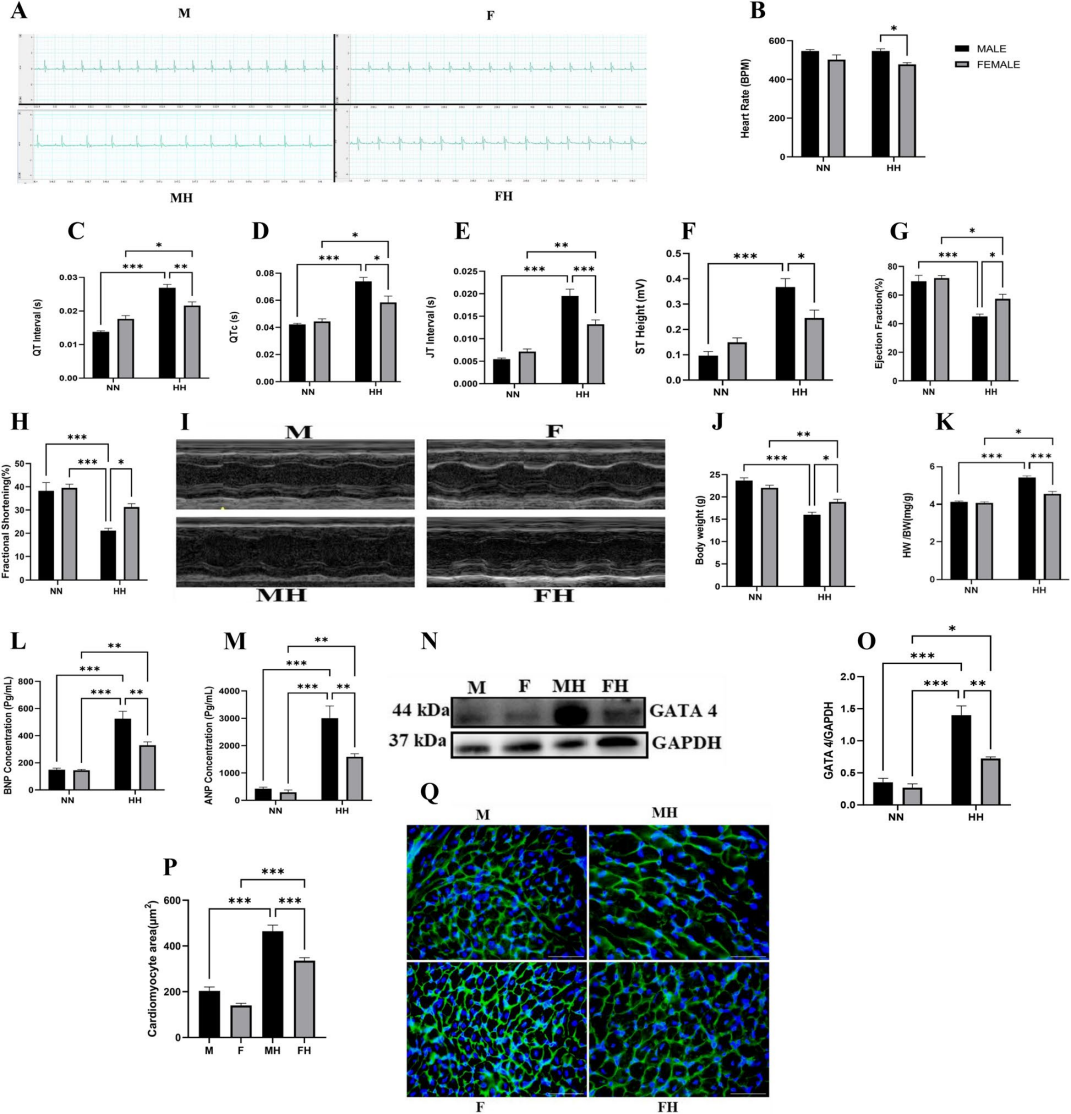
HH Induced Alteration of Cardiac Function with severity pronounced in male mice. **A**-**F** Representative electrocardiography (ECG) and Graphical presentation of ECG parameters including; heart rate, QT, corrected QT (QTc), JT Interval, and ST Height (*n* = 6–8/ mice per group). **G**-**I** Representative short-axis view M-mode echocardiogram imaging and graphical presentation of ejection fraction and shortening fraction parameters (*n* = 4). **J**-**K** Graphical presentations of morphometric data demonstrate alterations in the ratio of body weight/heart weight (HW/BW) and body weight (BW) (*n* = 5–6). **L-M** Effect of HH on plasma BNP and ANP concentration (*n* = 4–5). **N-O** Representative Immunoblotting of GATA4 and its Graph plot (*n* = 3 hearts per group). **P**-**Q** Representative Wheat germ agglutinin (WGA) staining and Graphical presentation of Cardiomyocyte surface area (*n* = 8–12 cells per section per 4–6 heart per group). M = male; MH = male + HH; F = female; FH = female + HH. Data were analyzed using Two-way ANOVA, followed by Turkey's multiple comparison test. ***p < 0.001; **p < 0.01; *p < 0.05

The electrocardiography of mice exposed to HH showed cardiac electric activity impairment compared to the NN mice. However, these effects were much more pronounced in male HH mice, which had increased heart rates (HR), a substantial prolongation of the QT interval, corrected QT interval and the JT interval, and an ST height compared to females (Fig. 1A-F). The significant alterations in these indexes indicate arrhythmias in the male mice. Also, the results obtained from systolic function assessment using echocardiography demonstrated that HH mice had left ventricular systolic alterations compared to NN mice. However, these effects were far more apparent in male mice, which showed a significant decrease in ejection fraction (EF) and fractional shortening (FS) compared to female mice under HH conditions (Fig. 1G-I).

### HH induced myocardial hypertrophy and injury with increased severity in males

Compared to the NN group, the male HH mice had a significantly reduced body weight with a corresponding increase in the HW/BW ratio (Fig. 1J-K). However, these effects were far more apparent in male HH mice. Additionally, increased cardiomyocyte area (Fig. 1P-Q), atrial natriuretic peptide (ANP), brain natriuretic peptide (BNP), and GATA4 expression were observed in male compared to female HH mice.

### Sex Difference in CD73 expression and activity during HH

**Fig. 2:**
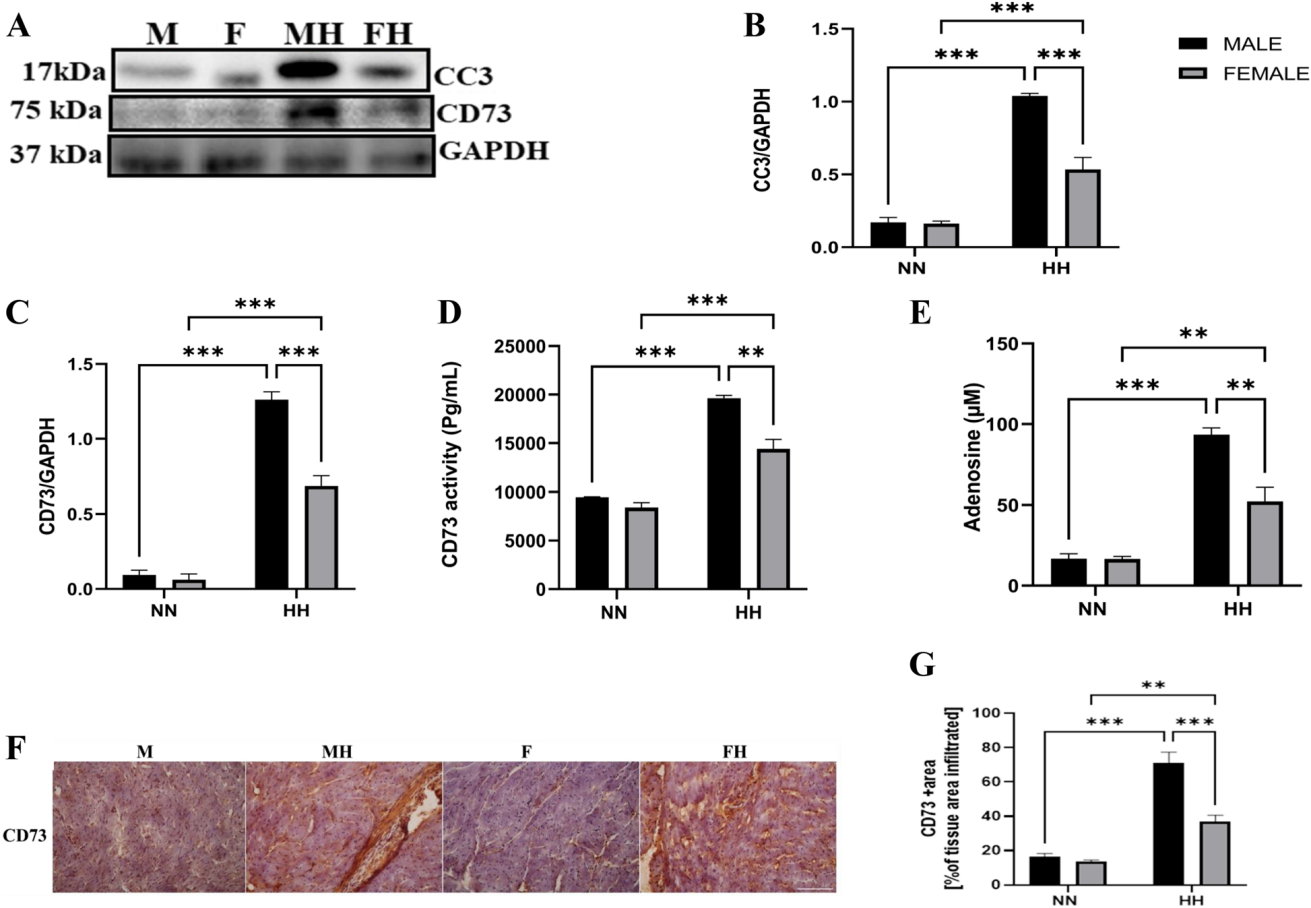
Sex difference on the CD73/adenosine axis. **A**-**C** Representative immunoblots, graphical presentations of CC3 and CD73 expressions (*n* = 3 mice/group). **D**-**E** ELISA analysis of Extracellular Adenosine level and CD73 activity (*n* = 4 mice/group). **F**-**G** Representative immunohistochemical staining and graphical presentation of CD73 expression from the myocardial sections (*n* = 4–5 hearts per group) Scale bar, 50 μm. M = male; MH = male + HH; F = female; FH = female + HH. Data were analyzed using Two-way ANOVA, followed by Tukey's post hoc analysis. ***p < 0.001; **p < 0.01; *p < 0.05

The expression and activity of CD73, along with adenosine concentration, were investigated to explore potential sex-specific variations in the CD73/adenosine axis. According to previous reports, apoptotic cells release substantial ATP into the extracellular space via pannexin 1, which is then converted to adenosine via CD39 and CD73 [27, 28]. Our results show that HH mice had elevated cleaved caspase 3 (CC3) and CD73 expression levels compared to NN mice, with increased CD73 activity and adenosine levels in both sexes. These differences were more pronounced under HH conditions in male mice (Fig. 2A-G), suggesting sex-specific variations in the CD73/adenosine axis under hypobaric hypoxic circumstances.

### CD73/adenosine axis exerts sex-dependent immunomodulation and metabolic response during HH

**Fig. 3:**
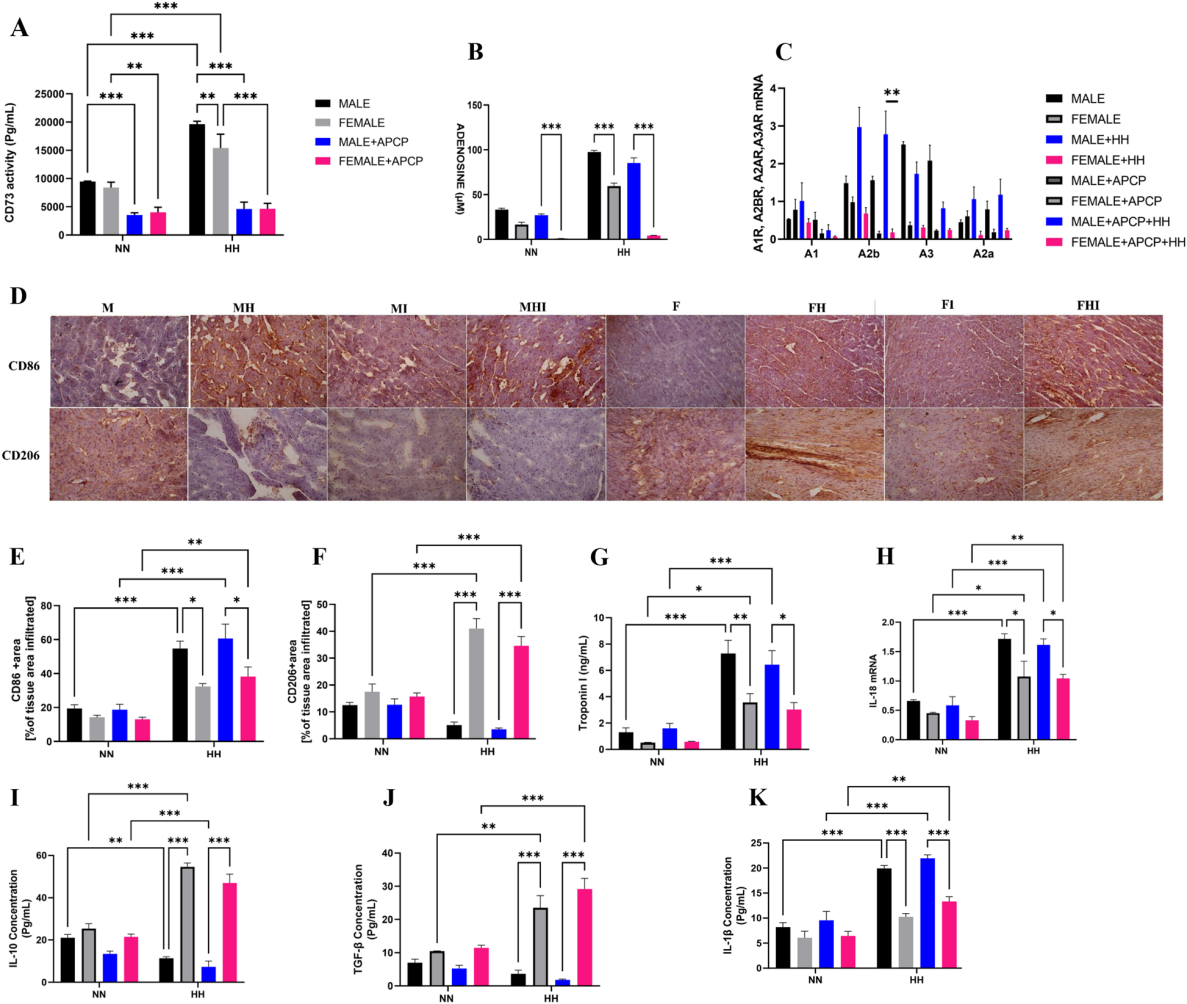
CD73/adenosine axis mediates immunomodulation during HH in a sex-dependent manner. **A**-**B** ELISA analysis of Extracellular Adenosine level and CD73 activity (*n* = 4 mice/group). **C** Relative mRNA levels of adenosine receptors (A1, A2a, A2b, A3 and *n* = 4mice/group). **D-F** Representative immunohistochemical staining and graphical presentation of macrophage CD86 and CD206-positive cells assessed from the myocardial sections (*n* = 4–6 hearts per group) Scale bar, 50 μm. **G-K** Graphical presentation of sera cardiac troponin I (cTnI) concentrations and inflammatory cytokines; Interleukin (IL)-1β, IL-18, IL-10, and transforming growth factor (TGF)-β concentrations assessed by ELISA and RT-qPCR using myocardia lysates. All ELISA were performed in triplicates (*n* = 5–6 mice per group). M = male; MH = male + HH; F = female; FH = female + HH; MI = male + APCP; FI = female + APCP; MHI = male + HH + APCP; FHI = female + HH + APCP. Data are presented as mean ± SEM; ***p < 0.001; **p < 0.01; *p < 0.05

Chronic hypoxia is reported to affect cardiac immunoregulation, with men displaying higher levels of inflammation [29–31]. The adenosine/CD73 axis is crucial for the immune response [32]. In this study, APCP administration in female mice (in normoxic (FI) or hypoxic environments (FHI)) decreased CD73 activity with a consequent decline in adenosine levels. Conversely, in male mice, APCP treatment (in normoxic (MI) or hypoxic environments (MHI)) resulted in only a decrease in CD73 activity without affecting adenosine levels (Fig. 3A-B). Similarly, among the four adenosine receptors, A2b expression remained elevated following APCP treatment in males but not females (Fig. 3C). Also, we found a significant influx of CD86 + (proinflammatory) macrophages into the myocardium of male mice (MH and MHI), whereas the CD206 + (anti-inflammatory) populations were reduced compared to females (FH and FHI). Additionally, the percentage of CD86 + macrophage infiltration in all groups was proportional to the plasma concentration of cardiac troponin I (cTnI) (Fig. 3D-G). Furthermore, evaluation of the levels of inflammatory cytokines in the hearts showed that proinflammatory cytokines (IL-1β and IL-18) were higher in male mice (MH and MHI) than in female mice (FH and FHI). In contrast, anti-inflammatory (IL-10 and TGF-β) protein levels were considerably lower in male mice (MH and MHI) than in female mice (FH and FHI) Fig. 3I-K. These findings suggest sex-dependent immunomodulation via the CD73/ adenosine axis during HH.

**Fig. 4:**
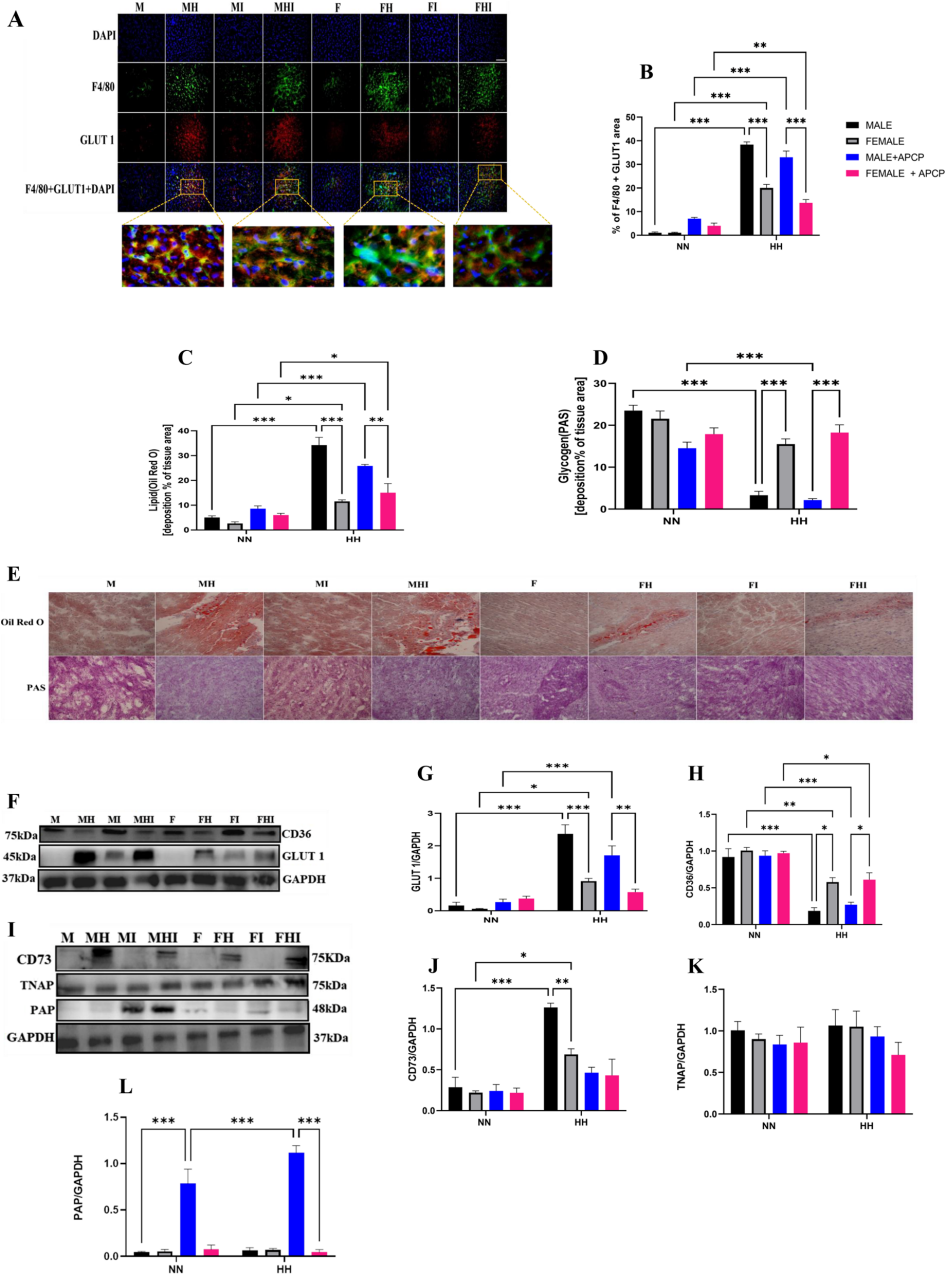
Hyperactivity of CD73/adenosine axis promotes myocardial metabolic shift in a sex-dependent manner during HH and protein expression of the other ectonucleotidases. **A**-**B** Representative Immunofluorescence and graphical presentation of the co-staining of F4/80 and Glut1 with nuclei (DAPI). Color channels were adjusted in the merged images to enhance the visualization of all the respective fluorescence dyes. Scale bar, 50 μm. **C**-**E** Representative Oil Red O (ORO) and Periodic Acid Schiff (PAS) staining of myocardial sections and their respective graphical presentations showing lipid and glycogen deposition percentages (*n* = 4–6 sections per 4–6 mice per group) Scale bar, 50 μm. **F**-**H** Representative Immunoblotting of CD36 and Glut1 and their respective Graphical plots; each blot band in the representative blot is an independent biological sample (*n* = 3 hearts per group). **I**-**L** Representative Immunoblotting of CD73, TNAP, and PAP and their respective Graphical plots; each blot band in the representative blot is an independent biological sample (*n* = 3 hearts per group). M = male; MH = male + HH; F = female; FH = female + HH; MI = male + APCP; FI = female + APCP; MHI = male + HH + APCP; FHI = female + HH + APCP. Data are expressed as mean ± SEM; *p < 0.05, **p < 0.01, ***p < 0.001

Chronic hypoxia induces a switch from lipid metabolism to glycolysis, which triggers immune cell reprogramming toward proinflammatory phenotypes and results in altered heart function [6]. We found an increase in macrophage GLUT1 expression under HH conditions related to NN in both sexes, with a significant increase in male mice (MH and MHI) compared to female mice (FH and FHI) under HH (Fig. 4A-B). Oil Red O and PAS analyses consistently revealed that HH mice had higher lipid accumulation than NN mice, whereas glycogen and other polysaccharides were lower. However, male mice (MH and MHI) under HH showed significantly lower glycogen and other polysaccharide contents and increased lipid accumulation (Fig. 4C-E). We further validated this occurrence using the fatty acid transporter (CD36) and glucose transporter protein (GLUT1) immunoblots. The findings showed that under HH, GLUT1 expression significantly increased, whereas CD36 expression decreased in males (MH and MHI) compared to females (FH and FHI) (Fig. 4F-H). These findings indicate that during HH, CD73/adenosine axis hyperactivity promotes macrophage reprogramming towards proinflammatory phenotypes in a sex-dependent manner.

### CD73 inhibition triggers PAP ectonucleotidase activity in male mice but not in females

To better understand the sex variations in the CD73/adenosine axis, immunoblots were used to assess the expression of certain compensatory enzymes that can also hydrolyze AMP to adenosine. Prostatic acid phosphatase (PAP) and tissue nonspecific alkaline phosphatase (TNAP) have been reported to degrade AMP to adenosine [33]. Here, we found that the protein expression of TNAP was unaffected in both male and female mice under HH or NN conditions. However, inhibition of CD73 activity during HH or NN significantly increased PAP protein expression in male mice (MI and MHI) but not in females (FH and FHI) (Fig. 4I-L). These findings suggest that PAP can sustain adenosine synthesis in CD73-inhibited male mice but not in female mice.

### CD73/PAP double inhibition reduced adenosine production and mitigated glycolytic shift and proinflammatory response

**Fig. 5:**
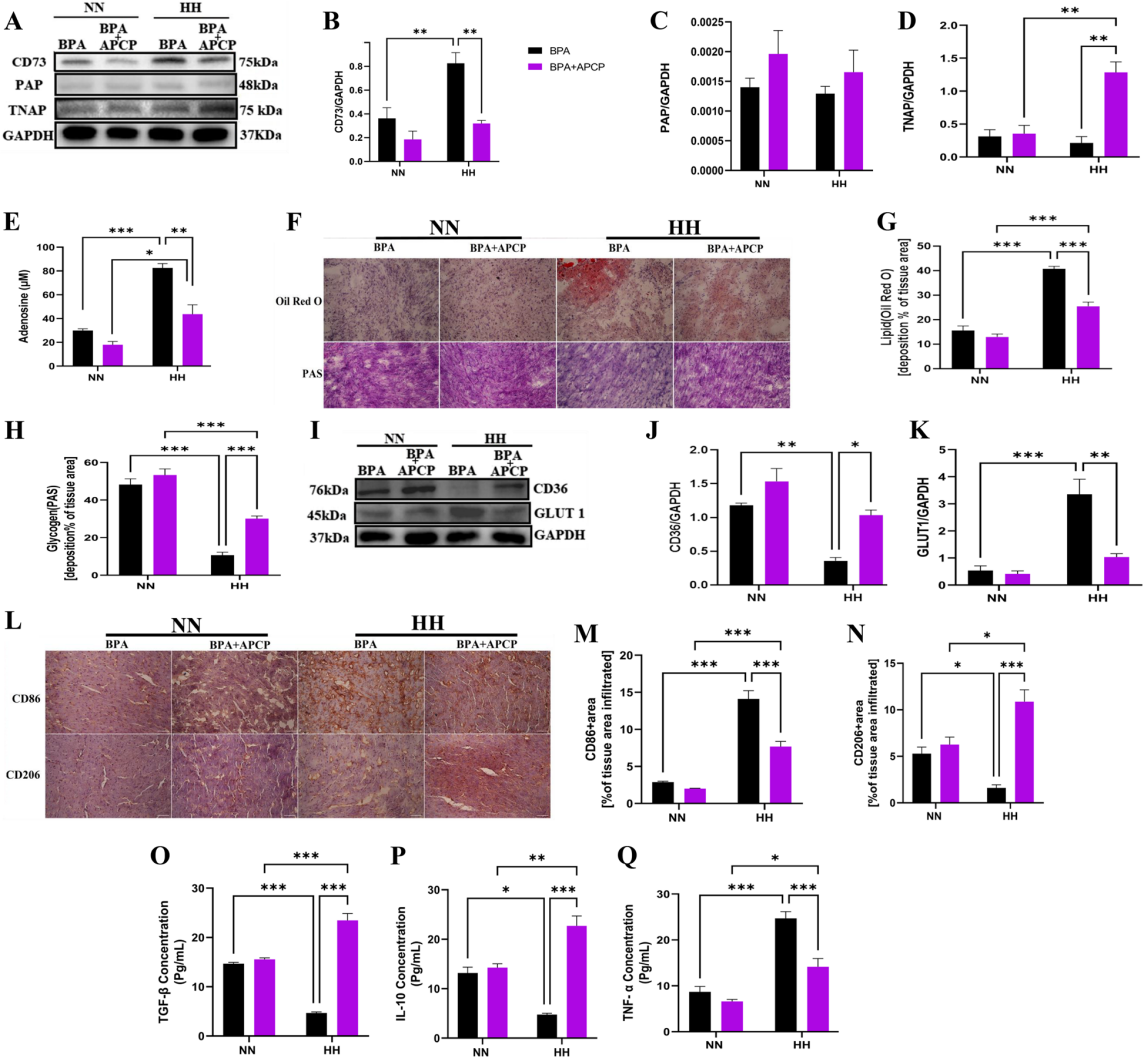
Double inhibition of PAP and CD73 in male mice, mitigated glycolytic shift and proinflammatory response. **A**-**C** Representative Immunoblotting of CD73, TNAP, and PAP and their respective Graphical plots; each blot band in the representative blot is an independent biological sample (*n* = 3 hearts per group). **E** ELISA analysis of Extracellular Adenosine level (n = 4 mice/group). **F**-**H** Representative Oil Red O (ORO) and Periodic Acid Schiff (PAS) staining of myocardial sections and their respective graphical presentations showing lipid and glycogen deposition percentages (*n* = 4–6 sections per 4–5 mice per group) Scale bar, 50 μm. **I**-**K** Representative Immunoblotting of CD36 and Glut1 and their respective Graphical plots; each blot band in the representative blot is an independent biological sample (*n* = 3 hearts per group). **L**-**N** Representative immunohistochemical staining and graphical presentation of macrophage CD86 and CD206-positive cells assessed from the myocardial sections (*n* = 4–5 hearts per group) Scale bar, 50 μm. **O**-**Q** Inflammatory cytokines; Interleukin TNF-α, IL-10, and transforming growth factor (TGF)-β concentrations assessed by ELISA using myocardia lysates. All ELISA were performed in triplicates (*n* = 4–5 mice per group). M = male; MH = male + HH; F = female; FH = female + HH; MI = male + APCP; FI = female + APCP; MHI = male + HH + APCP; FHI = female + HH + APCP. Data are expressed as mean ± SEM. *p < 0.05, **p < 0.01, ***p < 0.001

We further investigated the effect of double inhibition of PAP and CD73 on adenosine content. The results showed that a single inhibition of PAP activity with BPA decreased PAP expression but not adenosine content during NN or HH. In contrast, double inhibition of PAP and CD73 activity decreased PAP and CD73 expression and adenosine content to an optimal level. Furthermore, TNAP expression was significantly upregulated in mice with double inhibition of PAP and CD73 activity during HH (Fig. 5A-E). These results suggest that TNAP and PAP play synergistic roles, along with CD73, as the ectonucleotidases liable for converting AMP to adenosine in the hearts of male mice.

Oil Red O and PAS analyses also revealed that, compared to NN mice, HH mice had increased lipid deposition and reduced glycogen and other polysaccharide contents. However, single inhibition of PAP (BPA group) showed significantly lower glycogen and other polysaccharide contents and abundant lipid accumulation compared to the double inhibition group (APCP + BPA) under HH (Fig. 5F-H). Consistently, immunoblotting of the CD36 and GLUT1 proteins revealed that mice treated with BPA had significantly decreased CD36 expression during HH. In contrast, the expression of GLUT 1 significantly increased compared to the double inhibition (BPA + APCP) mice (Fig. 5I-K).

Finally, we found a significant influx of CD86 + (proinflammatory) macrophages into the myocardium of the BPA group, whereas the CD206 + (anti-inflammatory) population was reduced compared to the BPA + APCP group (Fig. 5L-N). Similarly, the proinflammatory cytokines (IL-1β and IL-18) were higher in the BPA group than in the BPA + APCP group under HH. Meanwhile, anti-inflammatory (IL-10 and TGF-β) proteins were lower in the BPA group compared to the BPA + APCP group under HH (Fig. 5O-Q). These findings suggest that CD73 and PAP inhibition mitigates glycolytic shift and proinflammatory response during HH by decreasing adenosine content to an optimum level.

## Discussion

The present study investigated the sex-dependent cardioprotective role of the CD73/Adenosine axis during hypobaric hypoxia. We observed an increased expression and activity of CD73 in both male and female mice during HH. However, this increase was remarkable in the male mice. Additionally, the hyperactivity of the CD73/adenosine axis recorded in male mice favored the switch from lipid metabolism to glycolysis, which drives macrophage reprogramming toward proinflammatory phenotype, leading to altered heart function. Also, we found that inhibition of CD73 with APCP caused an upregulation in the expression and activity of PAP protein, which sustained the adenosine content and failed to prevent glycolytic shift and proinflammatory response only in male (MI and MHI) mice. Lastly, the double inhibition of PAP and CD73 in male mice reduced the adenosine content to an optimum level, mitigating glycolytic shift and proinflammatory response.

Hypoxia is a potent modifier of the cardiovascular system [34]. It has been observed that after prolonged stay at high altitudes, some well-defined alterations occur in the heart [35]. For instance, during acute hypoxia, the sympathetic nervous system is activated to increase heart rate and systemic blood flow to preserve the function of both ventricles [36]. However, this adaptive mechanism is overwhelmed during prolonged exposure, resulting in decreased systemic blood flow despite an increased heart rate, leading to contractile dysfunction and left ventricle failure [4, 35, 37, 38]. Whereas intermittent hypoxia has been reported to be protective [39, 40], substantial evidence suggests that severe acute and prolonged exposure to high altitude precipitates maladaptive cardiac responses, which might be influenced by sex [41–45]. Consistent with these reports, we found that persistent exposure to HH significantly reduced the EF and FS of the heart despite the increased heart rate observed in male mice, indicating the occurrence of cardiac dysfunction. Also, our results show HH-induced prolongations of QT, QTc, JT, and ST height. Similar to previous reports [37, 38], these observations may be due to an increase in sympathetic activity during HH, causing prolonged repolarization, resulting in arrhythmia, cardiac failure, and sudden death [38]. The severity of these conditions was attenuated in female mice. Our results further support the evidence that sex-related differences exist in maladaptive cardiac responses to altitude/hypobaric hypoxia [41–45]. We also observed increased cardiomyocyte injury in male mice, characterized by elevated BNP and ANP levels and increased cardiomyocyte surface area compared to female mice. In line with our findings, Wearing et al. reported elevated BNP and ANP levels in male mice [46]. In addition, it has been demonstrated that a decrease in oxygen pressure led to increased synthesis of natriuretic peptides and that male mice were susceptible to higher cardiac natriuretic peptide levels compared to female mice during myocardial injury [47–49].

CD73/adenosine axis is vital for maintaining tissue integrity and facilitating recovery during myocardial damage [50]. We reported CD73/adenosine axis hyperactivity in male mice and a slight increase in females during HH. Also, we observed that the hyperactivity of the CD73/adenosine axis in male mice (MH) correlated with CC3 overexpression in male mice (Fig. 2A-G). Similar to our finding, it has been demonstrated that cells undergoing apoptosis release ATP into extracellular space via the pannexin 1 channel, which is later converted to adenosine by ectonucleotidases (CD39 and CD73) [51]. Moreover, myocyte apoptosis was reported to be more prominent in male mice [52, 53]. Sex differences in the CD73/adenosine axis have been reported in the brain and hepatocytes, with males showing increased expression and activity of CD73 under pathological state [19, 33]. Nonetheless, the sex difference in the CD73/adenosine axis during myocardial injury is unknown. Thus, we have shown here for the first time that sex difference exists in CD73 expression and activity during HH-induced myocardial injury.

Adenosine is an anti-inflammatory agent that prevents excess inflammatory reactions and is a therapeutic target for cardiac inflammation [54, 55]. We found that the overproduction of adenosine via the A_2B_ receptor during HH promoted the infiltration of proinflammatory macrophages (CD86 +) and increased proinflammatory cytokines in male mice (MH and MHI). In contrast, anti-inflammatory macrophages (206 +) infiltration and the concentration of anti-inflammatory cytokines were decreased. The opposite was observed in female mice under HH. In conformity with our finding, the overproduction of adenosine via the A_2B_ receptor was associated with elevation of proinflammatory cytokine, fibrosis, and organ damage [56–58]. Also, it has been reported that activation of inflammatory pathways can lead to myocarditis and that males are more susceptible to myocarditis than females [11, 59, 60].

Metabolic pathways utilized by activated immune cells significantly impact their immunological responses and functions [61]. Our results indicate increased GLUT1 expression in macrophages of male HH (MH and MHI) mice. Similarly, we observed increased GLUT 1 expression in the myocardium of male HH mice. This was associated with a substantial decrease in glycogen and other polysaccharide levels, whereas CD36 expression was reduced, correlating with a high level of lipid accumulation in male HH mice. These findings indicate a metabolic shift, which facilitates biased reprogramming of macrophages toward proinflammatory phenotypes, as previously reported [6, 62]. In addition, it has been reported that CD73 is a glycolysis-associated gene and is induced by hypoxia in cancer [63, 64]. Furthermore, our results show that, compared to female mice (FHI), male mice treated with APCP during HH (MHI) exhibited a decrease in CD73 activity despite having high adenosine levels (Fig. 3A-B). This suggests that inhibition of CD73 could not decrease adenosine overproduction and failed to prevent glycolytic changes and proinflammatory responses. Hence, it may be hypothesized that other enzymes, mainly expressed in male mice but not in females, characterize the conversion of AMP to adenosine. The existence of sex differences in CVDs has been primarily attributed to sex hormones such as estrogens. Estrogen is known to be a pleiotropy cardio-protective hormone [7, 65]. In our study, we noted that in the heart of female mice, the presence or the absence of the CD73/adenosine axis did not cause any significant alteration, possibly due to the pleiotropic cardio-protective function of estrogen, as we previously reported [28]. A study by Vasanthakumar et al. showed that testosterone administration in female mice caused an increase in the CD73/adenosine axis [66].

Adenosine is mainly generated by the degradation of ATP by ectonucleotidases (CD39 & CD73) [14]. Similar to CD73, TNAP and PAP are GPI-linked ectonucleotidases that can catalyze the conversion of 5′-AMP to adenosine [33, 67]. It has been demonstrated that TNAP and PAP can compensate for the loss of CD73 in the mouse brain and mediate the conversion of 5′-AMP to adenosine in the brains of CD73 − / − mice but not CD73 + / + animals [33, 67]. A similar observation was made in our study; we found that PAP protein expression was significantly upregulated in the male APCP mice (MI and MHI) group, while the protein expression of TNAP was unaffected compared to females (FI and FHI) (Fig. 4I-L). These results, therefore, confirm the hypothesis that other enzymes capable of converting AMP into adenosine may be present in male mice but not in females. The upregulation of PAP maintained excessive adenosine levels and failed to attenuate the glycolytic shift and proinflammatory responses in male mice. Hence, we posited that dual inhibition of PAP and CD73 could reduce adenosine levels and attenuate the glycolytic shift and proinflammatory responses in male mice under HH. As speculated, adenosine content was maintained at an optimal level, which mitigated glycolytic shift and proinflammatory responses in double-inhibition mic(BPA and APCP), compared to single-inhibited mice (BPA or APCP) groups under HH. Remarkably, we observed an upregulated TNAP in the double-inhibited male mice under HH (Fig. 5A-Q). Similarly, it has been reported that adenosine was partially generated in CD73 and PAP double KO mice, suggesting a third enzyme whose function was unmasked by PAP and CD73 inhibition [68]. This suggests that PAP and TNAP have a synergistic role with CD73 in the heart by contributing to the balance of extracellular adenosine production during pathophysiological events.

## Conclusion

The CD73/adenosine axis exhibits sex-dependent cardioprotection during HH. In the male heart, TNAP, PAP, and CD73 are the primary ectonucleotidases responsible for adenosine generation. To improve outcomes in pathological conditions associated with excessive adenosine production, provoking an inappropriate inflammatory response, simultaneous inhibition of PAP and CD73 proves cardioprotective by effectively regulating adenosine concentration. In contrast, female mice exclusively express CD73 as an ectonucleotidase, which, as demonstrated previously, safeguards the cardiovascular system during stress conditions, particularly when interacting with estrogens [28]. It is essential to acknowledge the limitations and translational implications of our findings. Our investigation did not explore other biological functions of TNAP. Notably, heightened TNAP protein expression has been linked to cardiomyocyte calcification despite earlier research highlighting its anti-inflammatory properties by converting proinflammatory ATP into the anti-inflammatory nucleotide adenosine [69]. Also, this present study employed chemical inhibitors instead of CD73 KO mice, so the results should be interpreted within that context. From a clinical translation perspective, our results underscore the significance of potential variations in CD73 enzyme expression levels, impacting the ATP/Adenosine ratio and thereby influencing the development of autoimmune diseases, either by stimulating or suppressing the immune response, as previously indicated [70, 71]. Additionally, the higher susceptibility of males to autoimmune myocarditis, possibly due to overproduction of the proinflammatory cytokines, has been reported [72–74]. Our findings show that excessive adenosine levels contributed to an elevated proinflammatory response, suggesting that increased myocarditis in males may stem from adenosine overproduction. Consequently, CVDs therapies targeting adenosine-generating enzymes should consider the involvement of other ectonucleotidases.

### Supplementary Information

**Supplementary Material 1.**

## Data Availability

The datasets generated in this study have been included in this article as figures.

## Abbreviations

BPA: Benzylphosphonic acid
HH: Hypobaric Hypoxia
APCP: CD73 inhibitor α, β-methylene adenosine-5'-diphosphate
NN: Normobaric Normoxia
TNAP: Tissue-nonspecific alkaline phosphatase
CVDs: Cardiovascular diseases
CC3: Caspase 3
PAP: Prostatic acid phosphatase
